# A novel technique for detecting sudden concept drift in healthcare data using multi-linear artificial intelligence techniques

**DOI:** 10.3389/frai.2022.950659

**Published:** 2022-08-31

**Authors:** Abdul Razak M. S., C. R. Nirmala, Maha Aljohani, B. R. Sreenivasa

**Affiliations:** ^1^Visvesvaraya Technological University, Belgaum, India; ^2^Department of Software Engineering, College of Computer Science & Engineering, University of Jeddah, Jeddah, Saudi Arabia

**Keywords:** financial data, concept drift, sliding window, random forest, data stream

## Abstract

A financial market is a platform to produce data streams continuously and around 1. 145 Trillion MB of data per day. Estimation and the analysis of unknown or dynamic behaviors of these systems is one the challenging tasks. Analysis of these systems is very much essential to strengthen the environmental parameters to stabilize society activities. This can elevate the living style of society to the next level. In this connection, the proposed paper is trying to accommodate the financial data stream using the sliding window approach and random forest algorithm to provide a solution to handle concept drift in the financial market to stabilize the behavior of the system through drift estimation. The proposed approach provides promising results in terms of accuracy in detecting concept drift over the state of existing drift detection methods like one class drifts detection (OCDD), Adaptive Windowing ADWIN), and the Page-Hinckley test.

## Introduction

A financial market is a place for trading where the buyers and sellers make their transactions. The financial market includes stocks, bonds, derivatives, foreign exchange, and commodities. The data from the financial market is now available in a stream fashion and the analysis of the data has to be done at run time. The users in the financial market use these analyzed results for the purchase of goods or to sell their goods (Yoo et al., 2005). A financial market is very dynamic and there are a lot of fluctuations due to environmental factors and also due to some hidden factors (Fdez-Riverola et al., 2007). The AI model developed to predict the financial market will become obsolete due to changes in the financial market. These changes have to identify and have to be informed to users for their intelligent trading. Concept drift is the term used to describe the target changes involved in data (Gama et al., 2014). If there is concept drift, then the model accuracy will decrease and the model misclassifies the data. Whenever a concept drift occurs in the data then we need to identify and update the model with recent data. In our work, we will address how to handle concept drift by monitoring the performance of the classifier using a sliding window, random forest algorithm, and Hoeffding decision tree for anytime classification of financialdata streams.

Concept drift can be categorized as (Gama et al., 2014):

**Table:** 

**Drift type**	**Behavior**	**Meaning**
Sudden, Incremental, Gradual and Recurrent		
Sudden	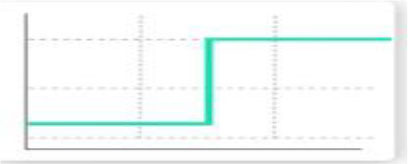	Changes quickly from one concept to another concept
Incremental	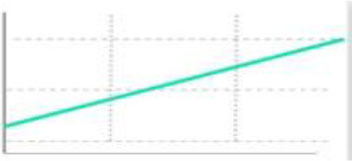	Changes happens slowly over time
Gradual	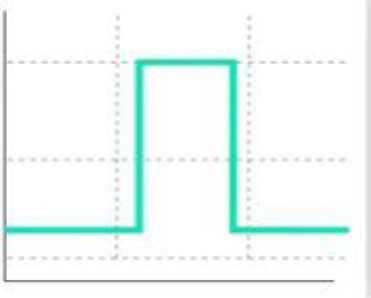	Concept diminishing with new one
Recurrent	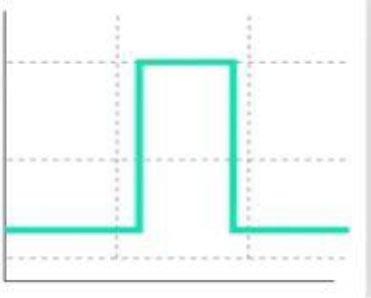	Concept repeats over time

Above Table 1 summarizes the different types error-based classification algorithms available to handle the different types of concept drift.

**Table 1:** Summary of Drift Detection algorithms (Firas et al., 2022).

**Category**	**Algorithm**	**Data retrieval**	**Test statistic calculation**	**Hypothesis test**	**Type of drift addressed**
Online error rate based	DDM (Gama et al., 2004)	Landmark window	Online error rate	Distribution estimation	Sudden drift
	EDDM (Baena-Garc'ia et al., 2006)	Landmark window	Online error rate	Distribution estimation	Gradual drift
	Page-Hinckley (Qahtan et al., 2015)	Sliding window	Average value	Performance means	Sudden drift
	ADWIN (Cavalcante and Oliveira, 2015)	Auto cut W_hist_, W_new_	Error rate difference	Hoeffding bound	Sudden / gradual
	OCDD (Gozuacik, 2021)	Sliding window	Percentage of outlier	*Post hoc* Neymenvi test	Sudden / gradual

There are five ways to deal with concept drift (Das, 2021):

- **Online learning:** The learner is regularly updated as the model processes each sample. Online learning is the most popular method for reducing concept drift in real-world applications.
- **Periodically retrain:** The model is activated when the model's performance falls below a predetermined level or when the average confidence score between two windows of data shows a significant drift.
- **Periodically re-train on a representative sub-sample:** The sample selects sub-samples from a large population in such a way that a portion of the sub-sampling sample represents the entire population. If concept drift is discovered, employ an instance selection strategy that employs the same probability distribution as the original data. Humans change the labels in the current dataset to fine-tune the model.
- **Ensemble learning with model weighting:** Multiple models are grouped together, and the weighted average of the individual model outputs is used as the overall output.
- **Feature dropping**: Another method for dealing with concept drift is feature dropping. Using a single feature, multiple models are built at the same time, and where the AUC-ROC response is inadequate, those features are dropped.

### Contribution of work

- A framework to detect concept drift in financial data streams by monitoring the performance of the model developed using a random forest algorithm and sliding window.
- Builds a decision tree incrementally using the Hoeffding tree for anytime classification and reset the tree once the drift is detected.
- Accuracy comparison of the proposed framework with one class drifts detection (OCDD), Page-Hinckley, and Adaptive Windowing (ADWIN) methods.
- Addresses the statistical significance of proposed framework using the Mcnemar's test.

### Organization of the paper

Chapter 1 gives the details about the introduction of our work. Chapter 2 gives the details about the literature methodology which will provide the essentials of our work. Chapter 3 addresses the background review of our topic which insights into the work carried out to detect concept drift in financial market data. Chapter 4 gives the process of our work i.e., the methodology we follow for the detection of concept drift. Chapter 5 provides the results of our work and comparison with the existing methods of drift detection. Chapter 6 gives the details about open research issues and research trends and chapter 7 details the future work to be done and chapter 8 gives the conclusion.

## Literature methodology

**Figure 1:**
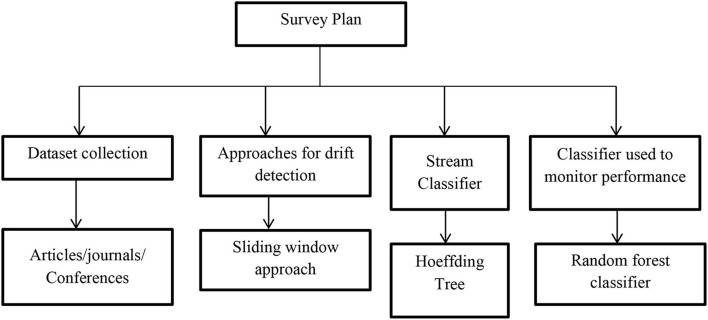
Literature methodology.

The survey framework designed for the literature is as shown in Figure 1. The literature review process involves the following horizons. Table 2 describes the extensive literature work carried out by different authors and also mentioned the limitations of their work.

- Data collection for financial market data.
- Data collection for sliding window and random forest classifier.
- Stream classifier for incremental tree building.

**Table 2:** Literature.

**Author**	**Title of the paper**	**Contributions**	**Limitations**
Gustavo H. F. M. Oliveira	Time Series Forecasting in the Presence of Concept Drift: A PSO-based Approach (Oliveira et al., 2017)	• Proposes Particle Swarm Optimization method to detect concept drift in time series financial data. • The proposed method is robust to false positive drift while maintaining low error rate during forecasting. • The experiment was conducted on four artificial datasets and three real time datasets from Dow Jones, NASDAQ and Yahoo finance. • The proposed method detects concept drift well compared to the state of the art methods like DDM, ECDD and FEDD.	• Proposed method based on swarm behavior (IDPSO-ELM-B) did not yield a good detection curve. • The methods ELMECDD and ELM-DDM monitor the error only for one model.
Bruno Silva	Applying Neural Networks for Concept Drift Detection in Financial Markets (Bruno and Nuno, 2012)	• Proposed a framework using neural networks to monitor the interday changes in financial stock market over the last 10 years of Dow Jones Industrial Average index (DJI). • The method comprises two phases i.e. online data aggregation using ART network and monitor error rate to detect concept drift using Average Quantization error. • The proposed method addresses gradual and abrupt drift in stock market data.	• The framework does not mention Intraday trading in financial stock market data streams.
Filippo Neri	Domain Specific Concept Drift Detectors for Predicting Financial Time Series (Filippo, 2021)	• Proposed three concept detectors myTanDD which uses angle between tangent to the data, MINPS uses data mean and minimum standard deviation of all data points, and mySD uses standard deviation to detect concept drift for financial time series data. • Data is collected in a sliding window to calculate the statistics and make a decision about concept drift. Hyper parameter tuning is considered to increase the performance of the proposed classifiers.	• Study of Hyper parameters tuning can impact the systems performance.
Hanen Borchani	Modeling Concept Drift: A Probabilistic Graphical Model Based Approach (Hanen, 2015)	• Propose a framework, based on probabilistic graphical models, that explicitly represents concept drift using latent variables. Data from a European bank from the period of April 2007 to March 2014 is considered. The proposed model finds the different trends in the economic climate and analyzed policies implemented by the BCC bank. The model finds the interesting concept drift information of streaming financial data and compared with other non-streaming techniques.	• Only one latent variable is used for modeling concept drift
Rodolfo C. Cavalcante	An Approach to Handle Concept Drift in Financial Time Series Based on Extreme Learning Machines and Explicit Drift Detection (Rodolfo, 2015)	• Proposed online sequential extreme learning machines (OS-ELM) with explicit drift detection algorithms to detect concept drift. It updates the model during the presence of concept drift. The proposed algorithm gives equivalent accuracy in forecasting the time series financial data and takes less time to detect the drift.	• During the negotiation in a real-world market, the intelligent trading system should consider concept drift.
J. Gama	Drift Detection Method (DDM) (Gama et al., 2004)	• Monitors the number of errors for detecting concept drift. • It has two levels to signal drift, warning and drift level. • Detects sudden drift only.	• Detection rate is low for different types of drift. • It monitors the error rate of the classifier.
Baena-Garcia	Early Drift Detection Method (EDDM) (Baena-Garc'ia et al., 2006)	• Early drift detection method (EDDM) based on the distance between the classification errors. • The early drift detection algorithm is able to detect the concept drift. When the gradual variations in the dataset are present then there is a chance of early detection.	• It uses two thresholds to warn and detect drift. • It monitors the error rate of the classifier.
Bifet	Adaptive Windowing (ADWIN) (Cavalcante and Oliveira, 2015)	• Proposed Adaptive-windowing (ADWIN) in which the window capacity is decided entirely by the rate of change seen in the data contained inside the window in the adaptive windowing approach. Here, a combination of NB and ADWIN supervises the error rate generated by the model and also makes the decision that the sample needs to be altered or not.	• ADWIN uses two sub-windows and compares changes in two sub windows. • It takes more computational time for deciding the sub window sizes.
A. A. Qahtan	Page-Hinckley Test (PHT) (Qahtan et al., 2015)	• Page hinckley test(PHT) employs statistical variation detection is employed to obtain the clusters for the data for detecting the drifts. For the model learning the DDM is employed and to detect the variation in the signal the PHT is used. • To detect the variation a continuous and a thorough examination is performed in PHT. By performing the average of the variations and distributions the concept drift can be detected.	• It uses two hypothesis tests to monitor the change in hypothesis to check for increase or decrease.
O. Gozuacik	One Class Drift Detection (OCDD) (Gozuacik, 2021)	• Implicit algorithm termed One-Class Drift Detector (OCDD) employs a one-class learner SVM and a window that slides to detect drift. • The classifier is trained to distinguish between the old and new instances and evaluate, if they are comparable. • If true, then indicates a drift depending on the rate of abnormality (outlier percentage) identified in the sliding window.	• Comparison of accuracy for the model by employing with different svm kernels. • Dataset is numerical in nature.
Tatiana Escovedo, Adriano Koshiyama, Andre Abs da Cruz, Marley Vellasco	DetectA: Abrupt Concept Drift Detection in Non-stationary Environments (Tatiana et al., 2018)	• DetectA is a concept drift detection method created for sudden concept drift detection. The primary innovation of this method is that it is proactive, as contrast to other drift detection approaches, which only identify concept drifts after they have already occurred. • A method for producing datasets with predefined sudden drifts has been suggested. In order to understand the degree of each parameter's influence on DetectA's ultimate performance, • A process based on differences in the amount of attributes, patterns, and imbalance rates between classes was used. • The detector is effective and appropriate for high-dimensional datasets, blocks of medium size, any amount of drifts, and class imbalance.	Clustering evaluation is not done using the metrics
Osama A.Mehdi, Eric Pardede, Nawfal Ali, Jinli Cao	Fast Reaction to Sudden Concept Drift in the Absence of Class Labels (Osama et al., 2020)	• A brand-new concept drift detector dubbed DMDDM-S that employs the PH test along with its computations to alter the disagreement measure. To determine the diversity of classifier responses in response to changing incoming data, DMDDM-S is proposed. • DMDDM-S uses the fading factor to track the diversity of a pair of classifiers instead of keeping track of the error estimates. • In comparison to the current methods, DMDDM-S identifies drifts with a smaller delay, less detection runtime, and less memory use.	The model was developed for semi supervised environment.

## Background review

### Methodology

**Figure 2:**
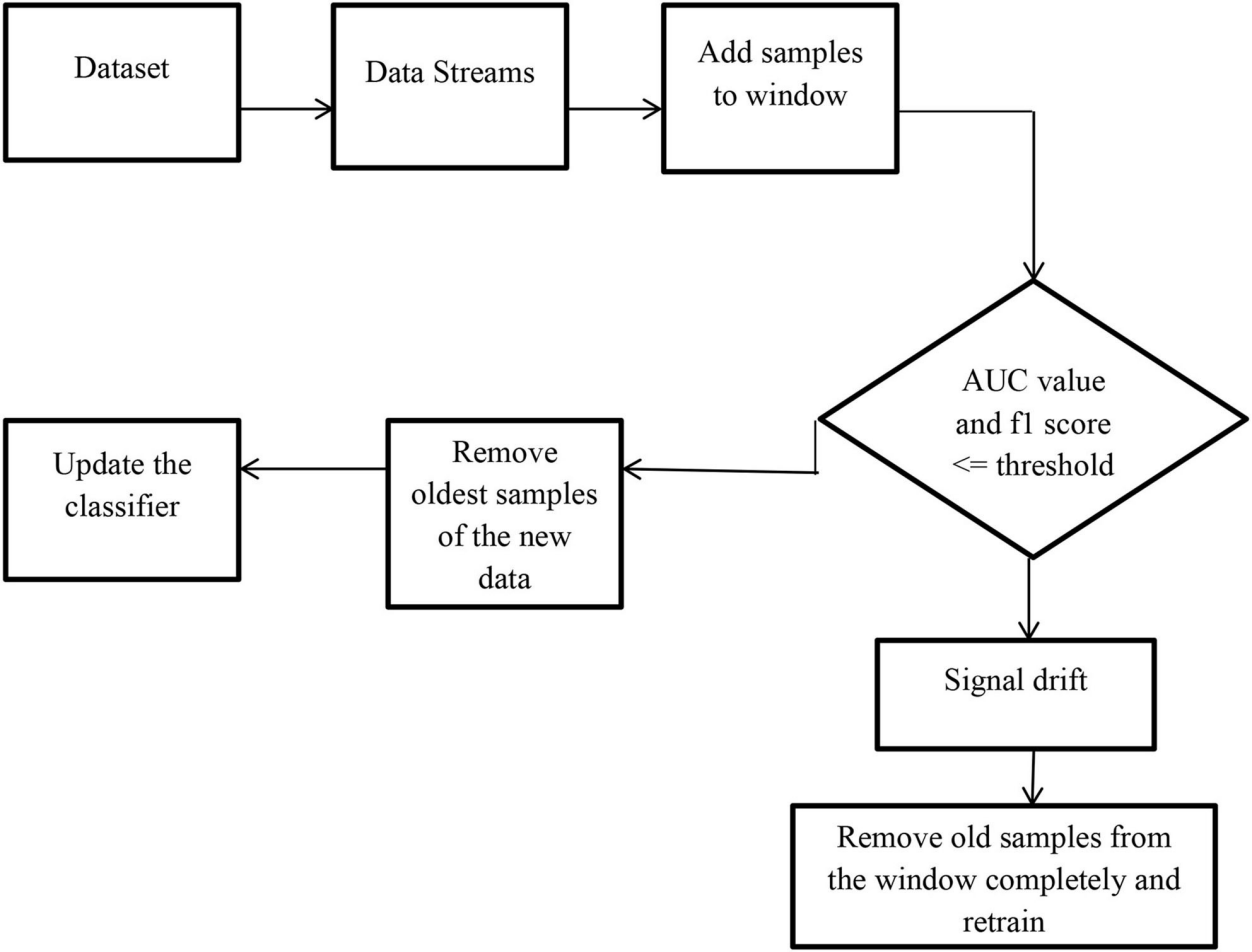
Framework to detect sudden concept drift in data streams.

As shown in Figure 2, the data blocks are read to the model in a streaming fashion [4] and the random forest algorithm is used to develop the AI model and the performance of the model is monitored through classification metrics. If the accuracy of the model is less than the threshold then the model is rebuilt over the new data. We read each instance in the window and start to build the Hoeffding tree incrementally using the Hoeffding stream classifier. Once the data in the window is full the window is subjected to a random forest algorithm to monitor the performance of the model. If the performance of the classifier is below the threshold value then concept drift is signaled and the current tree builds incrementally used for making decisions will be discarded and in the window, a new space will be made to fill out the new samples to reflect the current distribution.

### Algorithm

**Step 1:** Read data incrementally into the defined window size until the window becomes full.

**Step 2:** Train the model with the current window data using the Random forest algorithm and measure the performance of the model. If the performance of the model is less than the defined threshold then signal drift and go to step 3 else go to step 4.

**Step 3:** If there is a drift in the window data then remove the w^*^ ρ samples completely from the window and go to step 1.

**Step 4:** If there is no drift then remove w^*^(1- ρ) data samples from the window and go to step 1.

**Step 5:** If there are no samples remaining from the incoming data source then go to step 6.

**Step 6:** Exit.

### Pseudo code

**Table:** 

**Algorithm-:** Concept Drift Detector using Sequential analysis **Concept Drift Detector** (d, w, r, t): // d: Data Stream; w: window size; ρ: percentage of new data; t: threshold Window size S = (old data size + new data size) Stream classifier SC = Hoeffding Tree Classifier Drift Detection classifier DC = Random Forest algorithm **for** each instance in d **do** | Check IsEmpty(S) | **if** Yes **then** | | add instance X to window S | | Train model SC | **else** | | |S| = |T| // Combine data with class labels | | target = old for O [1, w] | | target = new for N [w+1, end] | | Train target with DC | | Measure the performance metrics | | Check Drift (DC, T) | | **if** Yes **then** | | | Shift (w^*^ ρ) old data from the window S | | | Reset and Retrain SC | | **else** | | | Shift w^*^(1- ρ) of old data w from the window S | | | and Train model SC | | **end** | **end** **end** IsEmpty(S): window index < window size Drift (DC,T): **if** AUC score and f1 score ≥ 0.7 **then** | drift = No **else** | drift = Yes **end**

## Results and discussions

### Dataset description

**Figure 3:**
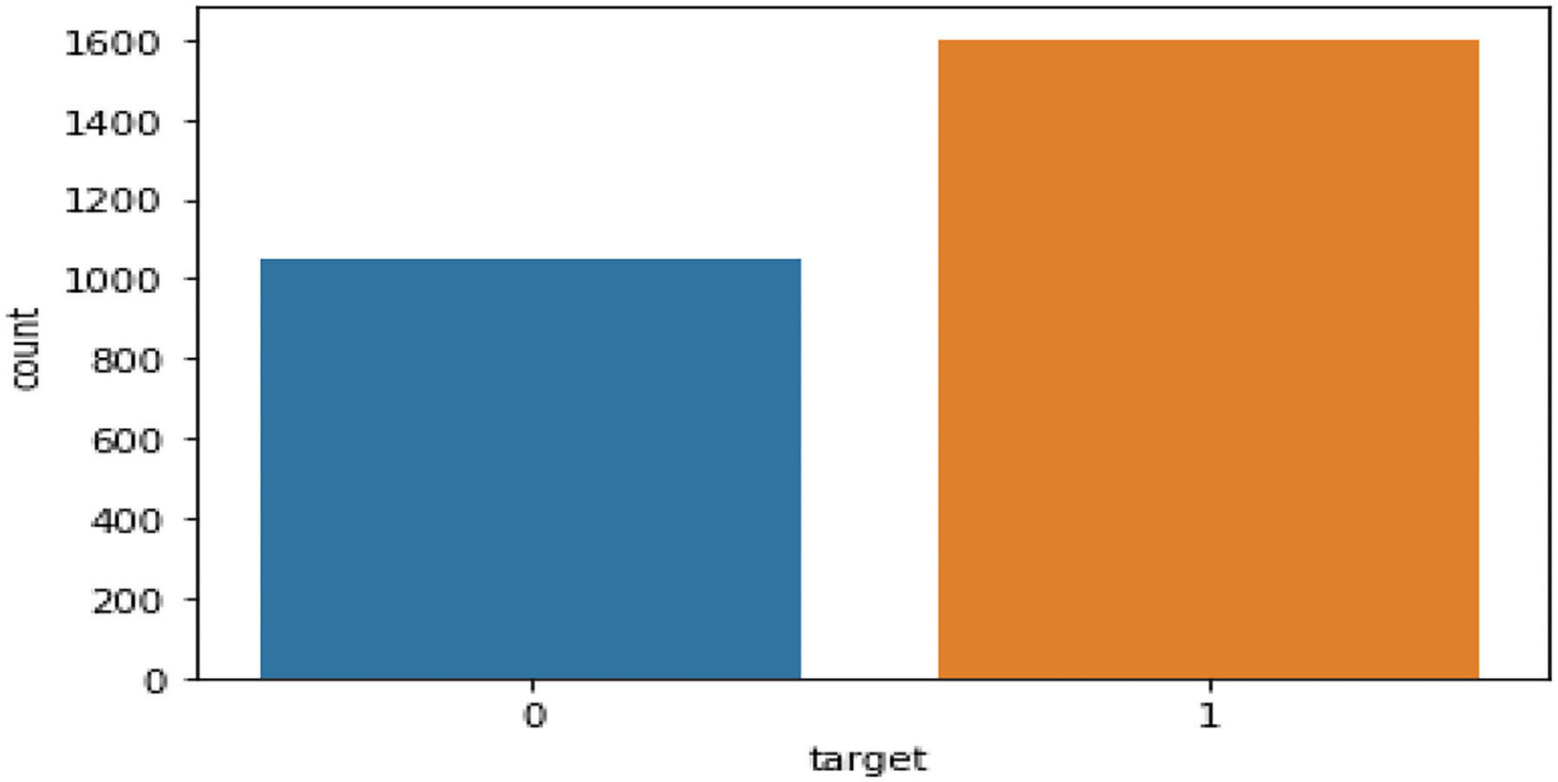
Data distribution of target variable.

The dataset characteristics is presented in Table 3. The data is collected weekly from the poll done by the American association of individual investors and the dataset contains information from January 1st, 2003 to December 31st' 2020 from three different countries China, India, and UAE. The dataset contains the description of the US sentiment investors, Measure of Variability (spread) and US returns collected weekly, social and cultural development indicators like Human development, Gross development and Population growth (yearly), and other Sectors–Value Added (VA) as % GDP in achieving UN SDG 3 (Health and Wellbeing) & SDG 8 (Growth & Economic Development) like Human Development Index (HDI), Current Health Expenditure (CHE) as a percentage of GDP, and per capita, health expenditure in constant US$ are all factors in the health sector, Macro-Economic factors like risk rate, foreign direct investment, GDP (annual growth) and Inflation and also includes economic crisis and pandemic events as shown in Figure 3.

**Table 3:** Dataset features overview [18-23].

**Attribute description**	**Name of the attributes**
US sentiment	Bullish, neutral, bearish, 8-week BMA
Measure of variability	Spread
US Returns	Market return for US
Human development indicator	Human development index-HDI
Gross national income	Per capita CHE $, CHE %GDP
Population growth annual %	POP-G annual %
Health sector - nutrition	Anemia
Technology sector	INTERNET%
GDP - industry sector	Industry VA-% GDP
Manufacturing sector	MFG-VA%GDP
Services sector	SER-VA%GDP
Agriculture, fishery, forestry (AFF) Sectors	AFF VA-%GDP
Peer reviewed journals	PRJ-R&D
Sector - entrepreneurs	SELF EMP-T%, SELF EMP-M%, SELF EMP-F%
Stocks traded value (%GDP)	STOCKS-TRADED VALUE (%GDP)
Stocks traded turnover domestic (%)	STOCKS-TRADED-TO-D (%)
Real interest rate%	Real Int. rate%
Foreign direct investment	FDI NI%GDP
GDP-annual growth	GDP-AG%
Inflation - annual %	INF-A%
Economic crises (EC) and pandemic event (PE)	EC-PE CODE

**Figure 4:**
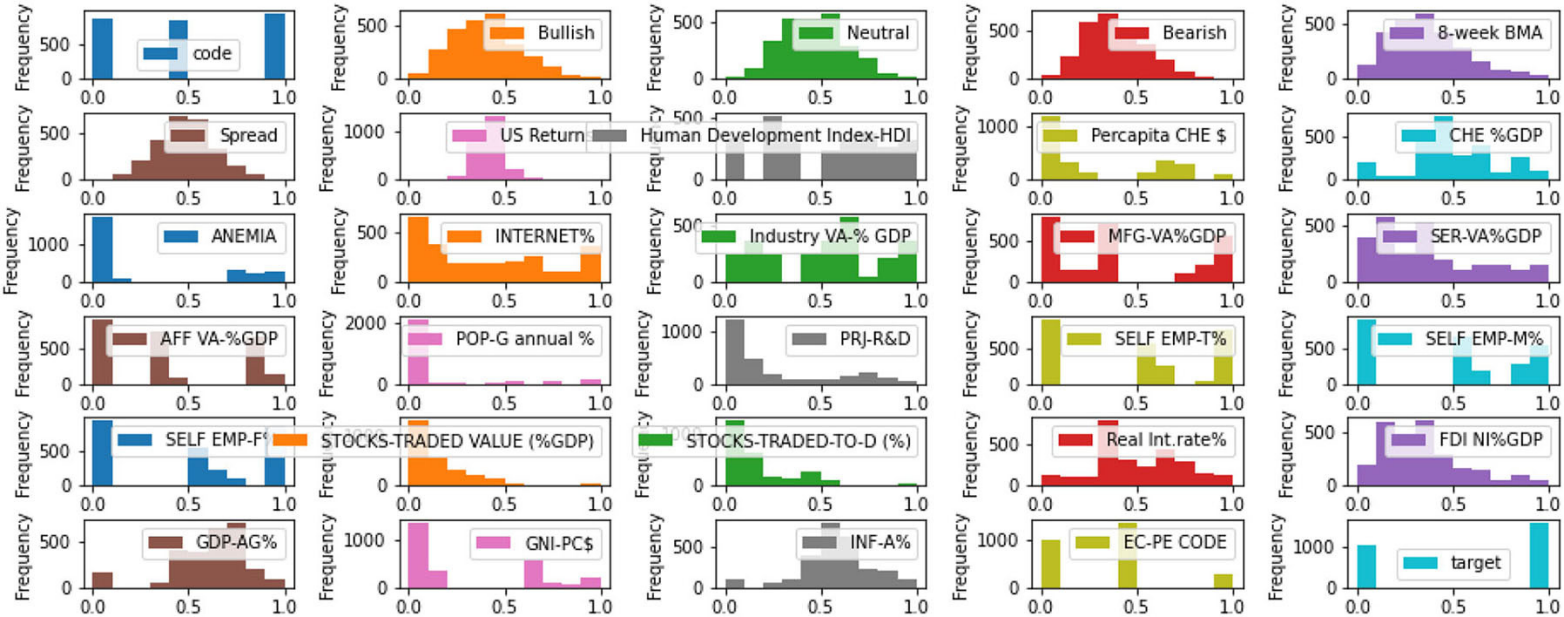
Data distribution of features.

The classification task is to tell whether the country (China, India, and UAE) is going to retain their investors every week. (1 - Yes and 0 - No) as shown in Figure 4. Table 3 describes the Dataset features^1^, ^2^, ^3^, ^4^, ^5^, ^6^, ^7^ used by the authors for their implementation.

### Classification metrics

**Table:** 

	**Predicted values**		
		0	1
**Actual values**	0	TN	FN
	1	FP	TP

TP = True Positive, FP = False Positive

TN = True Negative, FN = False Negative

Accuracy **=** TP + TN / TP + TN + FP + FN

Precision = TP / TP + FP

Recall = TP / TP + FN

F1-score = 2^*^precision^*^Recall / Precision + Recall

Macro-avg is the mean average of the F1 score of all classes.

Macro-avg = (F1 score of class 0 + F1 score of class 1) / 2.

Tables 4–6 describes the accuracy metrics for different data blocks. The weighted-average is calculated by taking the mean of all per-class F1 scores while considering each class's support.

**Table 4:** Classification report for Figure 5A TP = 116, FN = 64, FP = 69, TN = 76.

	**Precision**	**Recall**	**F1-score**	**Support**
0	0.54	0.52	0.53	145
1	0.63	0.64	0.64	180
Accuracy			0.59	325
Macro avg	0.58	0.58	0.58	325
Weighted avg	0.59	0.59	0.59	325

**Table 5:** Classification report for Figure 5B TP = 124, FN = 87, FP = 76, TN = 113.

	**Precision**	**Recall**	**F1-score**	**Support**
0	0.56	0.60	0.58	189
1	0.62	0.59	0.60	211
Accuracy			0.59	400
Macro avg	0.59	0.59	0.59	400
Weighted avg	0.59	0.59	0.59	400

**Table 6:** Classification report for Figure 5C TP = 129, FN = 116, FP = 83, TN = 147.

	**Precision**	**Recall**	**F1-score**	**Support**
0	0.56	0.64	0.60	230
1	0.61	0.53	0.56	245
Accuracy			0.58	475
Macro avg	0.58	0.58	0.58	475
Weighted avg	0.58	0.58	0.58	475

**Figure 5:**
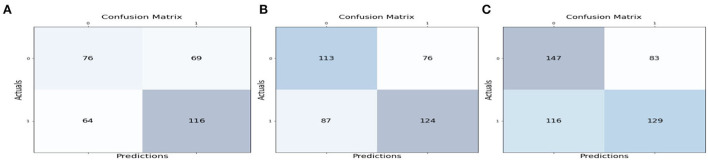
Confusion matrix values for the first three streaming blocks of data.

Example: Classification report for Figure 5A TP = 116, FN = 64, FP = 69, TN = 76

Macro Average = (F1 score of+ class 0 + F1 score of class 1) / 2.

= (0.53 +0.64) / 2 = 0.58

Weighted Average = Mean of all per-class F1 scores while considering each class's support.

= (0.53 ^*^ (145/325) + 0.64 ^*^ (180 /325))

= 0.59

Similar to Figure 5A, the calculation for macro average and weighted average will be done for 5B and C.

Table 7 displays the comparison bar graph between the proposed concept drift detection framework results and OCDD method results for different hyperparameter values like window size, threshold, and percentage of data in the sliding window. In comparison, the proposed framework gives good results for the accuracy metric over OCDD for smaller window sizes i.e. from window sizes 25 to 250.


$$\begin{array}{l} \begin{array}{l} \left[w=250,rho=0.1,T=0.9][w=250,rho=0.2,T=0.8\right]\\ \;[w=250,rho=0.3,T=0.7] \end{array} \end{array}$$

**Table 7:** Accuracy comparison of proposed concept drift detection technique with once class drift detection (OCDD) for different values of hyperparameters like window size, percentage of new data, and threshold.

**Threshold = 0.7, Percentage of new data = 0.3**	** 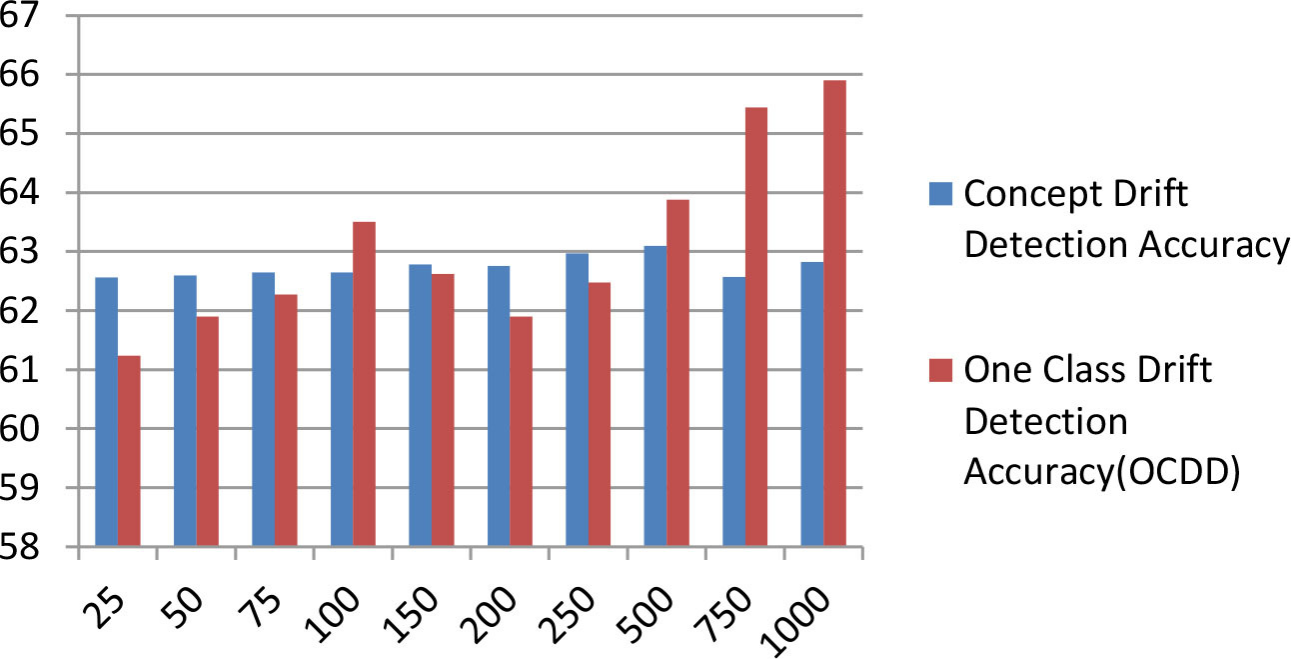 **
**Threshold** **=** **0.8, Percentage of new data** **=** **0.2**	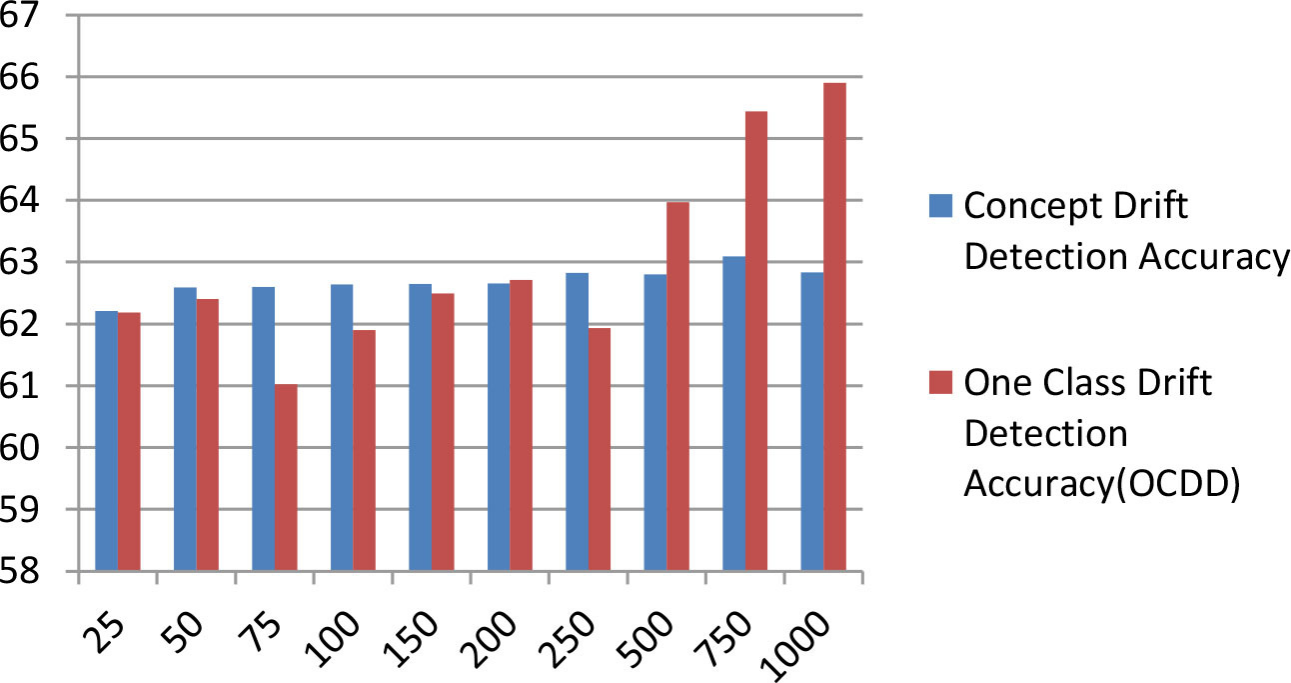
**Threshold** **=** **0.9, Percentage of new data** **=** **0.1**	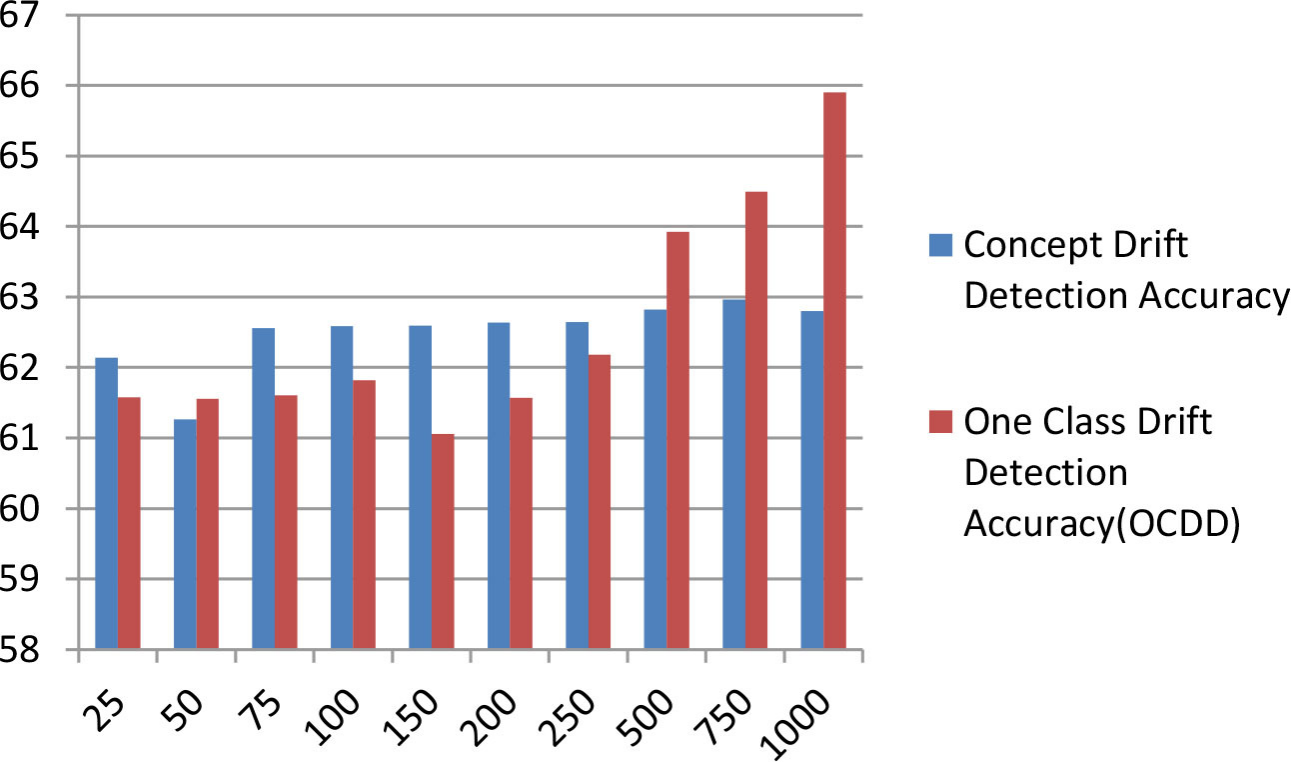

x-axis represents window size and the y-axis represents accuracy in percentage.

**Figure 6:**
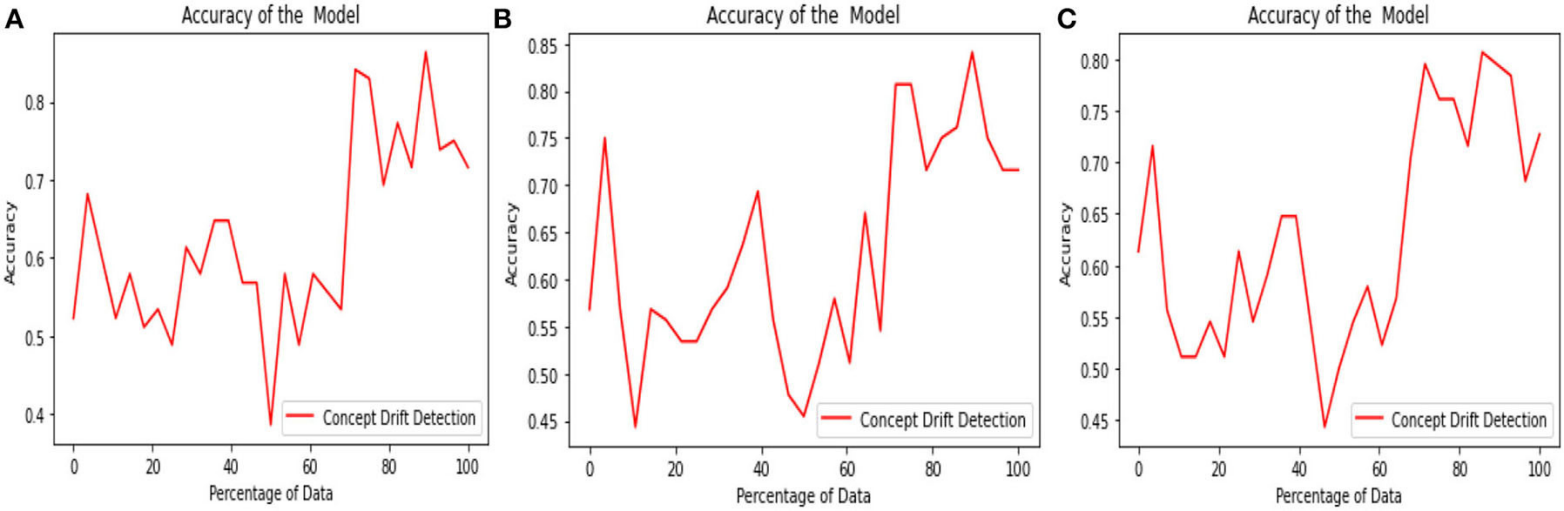
Accuracy Graph of proposed concept drift detection technique for window size =250, percentage of new data = [0.1, 0.2, and 0.3] and threshold = [0.9, 0.8, and 0.7].

The above Figures 6A–C diagrams depict the accuracy graph of the proposed concept drift detection technique for different hyperparameter values. The x-axis displays the percentage of data and the y-axis displays the accuracy. Whenever the accuracy of the model declines below 0.7 then concept drift will be signaled and the percentage of data will be added to the sliding window.

**Figure 7:**
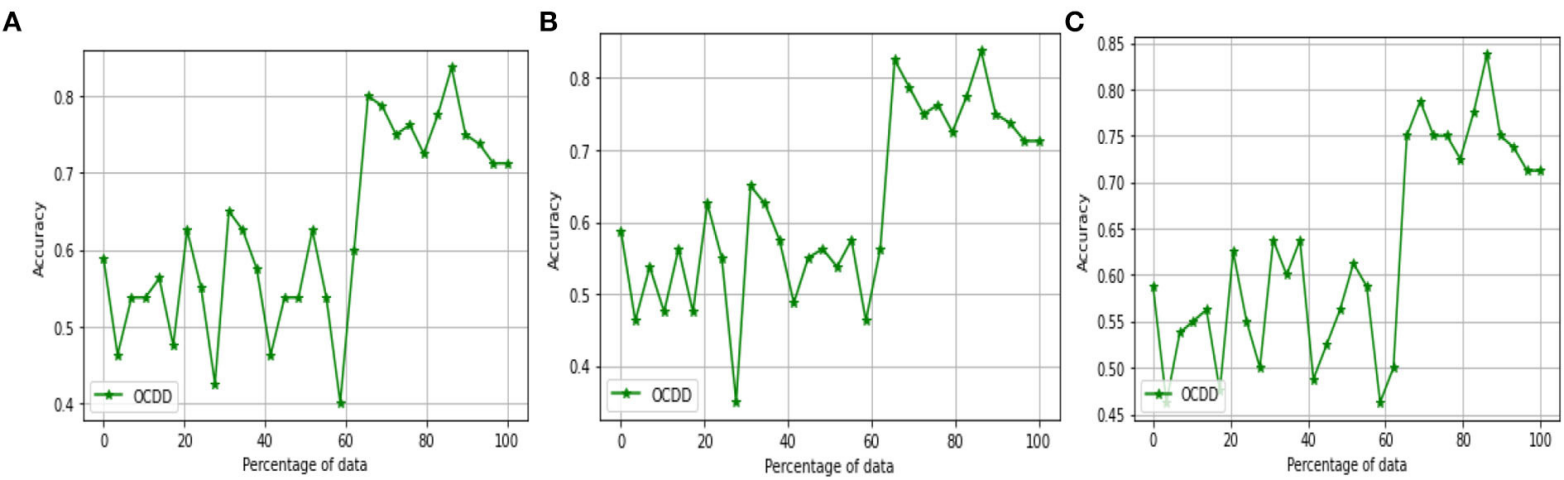
Accuracy Graph of one class drift detection technique [OCDD] for window size =250, percentage of new data = [0.1, 0.2, and 0.3] and threshold = [0.9, 0.8, and 0.7].

The above Figures 7A–C diagrams depict the accuracy graph of one class drift detection technique for different hyperparameter values. The x-axis displays the percentage of data and the y-axis displays the accuracy.

**Figure 8:**
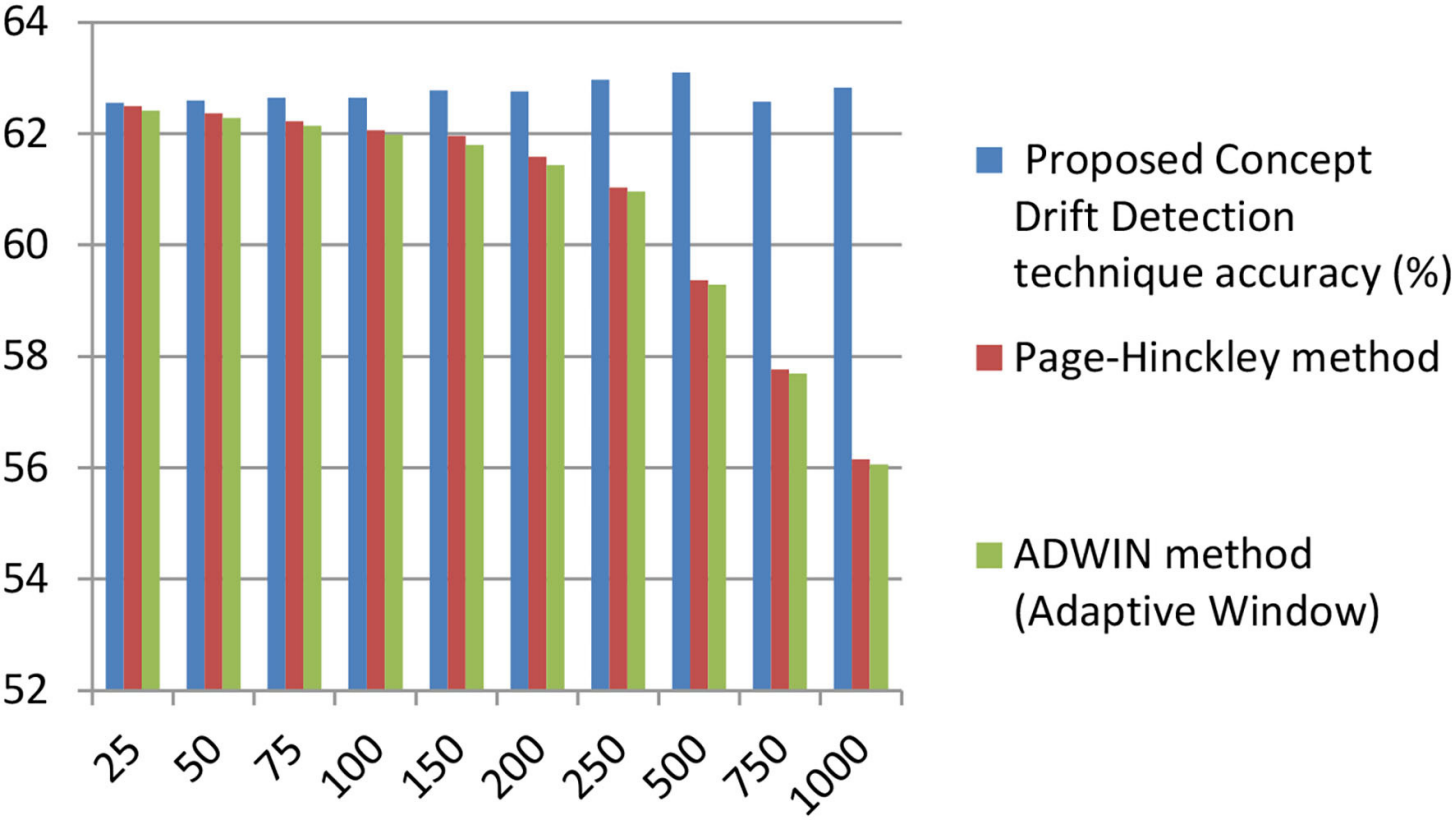
Accuracy comparison of proposed concept drift detector technique with Page –Hinckley, and ADWIN (adaptive window) method for varying window size. (x-axis represents window size and the y-axis represents accuracy).

Figure 8 depicts the comparison of the proposed concept drift detector technique with the Page-Hinkley method and window-based method ADWIN. In comparison, the proposed method outstands in accuracy for different values of window size.

A random forest algorithm is used in the proposed solution to develop the AI model and to monitor the performance. We have tuned the tree depth to create an appropriate balance between bias and variance to get the optimum generalization performance.

The following Tables 8–10 describe the tuning of the tree depth for the window size w =250 for threshold ε {0.7, 0.8, 0.9} and percentage of new data (ρ)ε {0.3, 0.2, 0.1}

**Table 8:** Window size = 250, Threshold = 0.7 Percentage of new data = 0.3.

**Depth**	**Number of estimators**	**Minimum samples leaf**	**Maximum features**	**Precision**	**Recall**	**F score**	**Accuracy**
05	100	50	Auto	61.4405	62.9658	61.0087	62.9658
10	100	50	Auto	61.4405	62.9658	61.0087	62.9658

**Table 9:** Window size = 250, Threshold = 0.8 Percentage of new data = 0.2.

**Depth**	**Number of estimators**	**Minimum samples leaf**	**Maximum features**	**Precision**	**Recall**	**F score**	**Accuracy**
05	100	50	Auto	61.3117	62.8220	60.8498	62.8220
10	100	50	Auto	61.3117	62.8220	60.8498	62.8220

**Table 10:** Window size = 250, Threshold = 0.9 Percentage of new data = 0.1.

**Depth**	**Number of estimators**	**Minimum samples leaf**	**Maximum features**	**Precision**	**Recall**	**F score**	**Accuracy**
05	100	50	Auto	61.1582	62.6428	60.7824	62.6428
10	100	50	Auto	61.1582	62.6428	60.7824	62.6428

**Figure 9:**
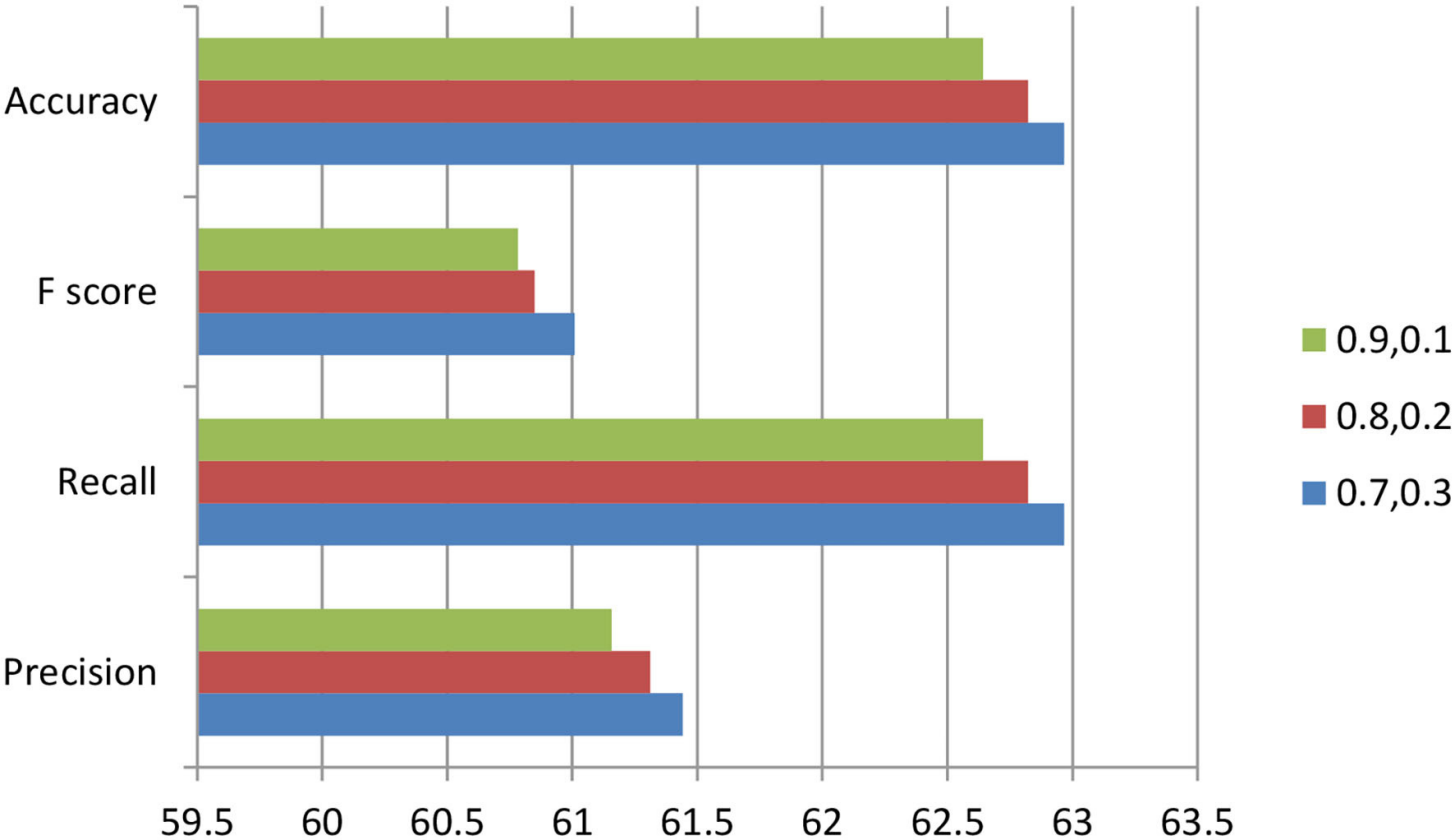
For Window Size =250, Threshold ∈ {0.9, 0.8, 0.7}, Percentage of new data ∈ {0.1, 0.2, and 0.3}.

Tuning the hyperparameters of the random forest like depth of tree ε {05, 10}, a number of estimators ε {100, 200}, the minimum number of samples in leaf node ε {50,100}, we found that for window size 250 the classification metrics will provide the promising results for threshold value = 0.7 and percentage of new data = 0.3 compared to different values of threshold and percentage of new data as shown in Figure 9.

We use Mcnemar's test to perform a significance test for classification to compare the accuracy of our proposed concept drift technique with the accuracy of the OCDD technique. The McNemar's test is a paired nonparametric or distribution-free statistical hypothesis test. It is used to test the significance of two classifiers over a single dataset. In the Mcnemar's test, the null hypothesis we formulate is that the performance of two models is the same, and in the alternative hypothesis that the performance of two models is different.

The McNemar's test^8^ statistic (“chi-squared”) can be computed as follows


$$\begin{array}{l} \chi^{2}=\frac{{\left(b-c\right)}^{2}}{\left(b+c\right)}-------\to \left(Equation\;1\right) \end{array}$$

With one degree of freedom and an alpha value of 0.05, we compute the *p*-value for some blocks in the below table.

**Table:** 

**Streaming blocks of data**		**p**	**Significance**
Block 1	Proposed method v/s OCDD	0.7287	True
Block 2	Proposed method v/s OCDD	0.4335	True
Block 3	Proposed method v/s OCDD	0.8180	True

## Open research issues and research trends

### Research issues

The following are some of the research issues that can be addressed in the future:

- Handling outliers and class imbalance in data streams during concept drift detection.
- To design a single drift classifier that can address all types of drifts.
- The majority of methods rely too heavily on tracking the decline in learner accuracy. To have a stronger assumption on drift detection, a multiple hypothesis technique could be used in conjunction with other metrics being monitored.

### Research trends

- To create data streaming techniques that scale to massive deep learning networks and are effective across all domains.
- Conducting online learning by utilizing distributed streaming engines, such as Apache Spark, Apache Flink, Apache Storm, and others, will be a key trend when dealing with massive amounts of data.
- Traditional deep learning methods must make numerous passes through the data. How to create models for concept drift detection in data streams that simply perform one pass through the data without saving the data.
- Unsupervised methods for handling concept drift in the absence of class labels.

## Future enhancement

The proposed work employs a framework for the detection of concept drift in financial data streams. The data employed in the framework for concept drift detection is numerical in nature and in the future can be worked on categorical data for concept drift detection. The framework is developed for sudden concept drift and can be used and analyzed for different types of drift. Multiple real-world and synthetic financial datasets can be considered for analyzing the results of the proposed framework. The time complexity of the model can be studied as a future scope.

## Conclusion

The proposed framework uses a random forest algorithm to detect sudden concept drift by monitoring the performance of the classification metrics like f1 score and AUC value with different threshold values for financial data streams. The proposed work detects sudden concept drift well for smaller window sizes and the results are compared with OCDD, Page-Hinckley, and ADWIN methods.

## Data availability statement

The original contributions presented in the study are included in the article/Supplementary material, further inquiries can be directed to the corresponding author.

## Author contributions

MA and CRN made substantial contribution to conception and design and acquisition of data. MA and SBR involved in analysis and interpretation of data. MA, CRN, and SBR drafted the article. MSAR contributed during the entire revision by answering to the reviewer comments and analysis of the proposed model results since from the first review process. All authors contributed to the article and approved the submitted version.

## Conflict of interest

The authors declare that the research was conducted in the absence of any commercial or financial relationships that could be construed as a potential conflict of interest.

## Publisher's note

All claims expressed in this article are solely those of the authors and do not necessarily represent those of their affiliated organizations, or those of the publisher, the editors and the reviewers. Any product that may be evaluated in this article, or claim that may be made by its manufacturer, is not guaranteed or endorsed by the publisher.

## Footnotes

^1^. “Multiflow,” scikit, June 17, 2020, https://scikit-multiflow.github.io/.

^2^. Assets.kpmg. https://assets.kpmg/content/dam/kpmg/ae/pdf-2020/09/uae-healthcare-perspectives.pdf.

^3^. “Investment Opportunities in China's Healthcare Sector after COVID-19.” China Briefing News, 26 Mar. 2020, https://www.china-briefing.com/news/investment-opportunities-chinas-healthcare-sector-aftercovid-19/.

^4^. “China Stock Market - Shanghai Composite Index.” MacroTrends, https://www.macrotrends.net/2592/shanghai-composite-index-china-stock-market-chart-data.

^5^. “Healthcare Industry in India, Indian Healthcare Sector, I...” Industry in India, Indian Healthcare Sector, Invest..., https://www.investindia.gov.in/sector/healthcare.

^6^. Healthcare July 2019 - IBEF. https://www.ibef.org/download/Healthcare-July-2019.pdf.

^7^. “Dubai: Global Healthcare Destination.” Medical Toursim, https://www.medicaltourism.com/destinations/dubai.

^8^http://rasbt.github.io/mlxtend/user_guide/evaluate/mcnemar/.
