# Supplementary material for: A novel technique for detecting sudden concept drift in healthcare data using multi-linear artificial intelligence techniques
Source: Front Artif Intell. 2022 Aug 31;5:950659. doi: 10.3389/frai.2022.950659 (PMC9471369; doi:10.3389/frai.2022.950659)
Supplement: Supplementary file 1 [file Data_Sheet_1.docx]

Outcome values:

TP = 116, FN = 64, FP = 69, TN = 76

|  | precision | recall | f1-score | support |
| --- | --- | --- | --- | --- |
| 0 | 0.54 | 0.52 | 0.53 | 145 |
| 1 | 0.63 | 0.64 | 0.64 | 180 |

| accuracy |  |  | 0.59 | 325 |
| --- | --- | --- | --- | --- |
| macro avg | 0.58 | 0.58 | 0.58 | 325 |
| weighted avg | 0.59 | 0.59 | 0.59 | 325 |

Outcome values:

TP = 124, FN = 87, FP = 76, TN = 113

|  | precision | recall | f1-score | support |
| --- | --- | --- | --- | --- |
| 0 | 0.56 | 0.60 | 0.58 | 189 |
| 1 | 0.62 | 0.59 | 0.60 | 211 |

| accuracy |  |  | 0.59 | 400 |
| --- | --- | --- | --- | --- |
| macro avg | 0.59 | 0.59 | 0.59 | 400 |
| weighted avg | 0.59 | 0.59 | 0.59 | 400 |

Outcome values:

TP = 129, FN = 116, FP = 83, TN = 147

|  | precision | recall | f1-score | support |
| --- | --- | --- | --- | --- |
| 0 | 0.56 | 0.64 | 0.60 | 230 |
| 1 | 0.61 | 0.53 | 0.56 | 245 |

| accuracy |  |  | 0.58 | 475 |
| --- | --- | --- | --- | --- |
| macro avg | 0.58 | 0.58 | 0.58 | 475 |
| weighted avg | 0.58 | 0.58 | 0.58 | 475 |

Outcome values:

129 149 83 189

|  | precision | recall | f1-score | support |
| --- | --- | --- | --- | --- |
| 0 | 0.56 | 0.69 | 0.62 | 272 |
| 1 | 0.61 | 0.46 | 0.53 | 278 |

| accuracy |  |  | 0.58 | 550 |
| --- | --- | --- | --- | --- |
| macro avg | 0.58 | 0.58 | 0.57 | 550 |
| weighted avg | 0.58 | 0.58 | 0.57 | 550 |

Outcome values:

129 187 83 226

|  | precision | recall | f1-score | support |
| --- | --- | --- | --- | --- |
| 0 | 0.55 | 0.73 | 0.63 | 309 |
| 1 | 0.61 | 0.41 | 0.49 | 316 |

| accuracy |  |  | 0.57 | 625 |
| --- | --- | --- | --- | --- |
| macro avg | 0.58 | 0.57 | 0.56 | 625 |
| weighted avg | 0.58 | 0.57 | 0.56 | 625 |

Outcome values:

129 217 83 271

|  | precision | recall | f1-score | support |
| --- | --- | --- | --- | --- |
| 0 | 0.56 | 0.77 | 0.64 | 354 |
| 1 | 0.61 | 0.37 | 0.46 | 346 |

| accuracy |  |  | 0.57 | 700 |
| --- | --- | --- | --- | --- |
| macro avg | 0.58 | 0.57 | 0.55 | 700 |
| weighted avg | 0.58 | 0.57 | 0.55 | 700 |

Outcome values:

129 252 83 311

|  | precision | recall | f1-score | support |
| --- | --- | --- | --- | --- |
| 0 | 0.55 | 0.79 | 0.65 | 394 |
| 1 | 0.61 | 0.34 | 0.44 | 381 |

| accuracy |  |  | 0.57 | 775 |
| --- | --- | --- | --- | --- |
| macro avg | 0.58 | 0.56 | 0.54 | 775 |
| weighted avg | 0.58 | 0.57 | 0.54 | 775 |

Outcome values:

153 271 93 333

|  | precision | recall | f1-score | support |
| --- | --- | --- | --- | --- |
| 0 | 0.55 | 0.78 | 0.65 | 426 |
| 1 | 0.62 | 0.36 | 0.46 | 424 |

| accuracy |  |  | 0.57 | 850 |
| --- | --- | --- | --- | --- |
| macro avg | 0.59 | 0.57 | 0.55 | 850 |
| weighted avg | 0.59 | 0.57 | 0.55 | 850 |

Outcome values:

201 271 120 333

|  | precision | recall | f1-score | support |
| --- | --- | --- | --- | --- |
| 0 | 0.55 | 0.74 | 0.63 | 453 |
| 1 | 0.63 | 0.43 | 0.51 | 472 |

| accuracy |  |  | 0.58 | 925 |
| --- | --- | --- | --- | --- |
| macro avg | 0.59 | 0.58 | 0.57 | 925 |
| weighted avg | 0.59 | 0.58 | 0.57 | 925 |

Outcome values:

250 271 146 333

|  | precision | recall | f1-score | support |
| --- | --- | --- | --- | --- |
| 0 | 0.55 | 0.70 | 0.61 | 479 |
| 1 | 0.63 | 0.48 | 0.55 | 521 |

| accuracy |  |  | 0.58 | 1000 |
| --- | --- | --- | --- | --- |
| macro avg | 0.59 | 0.59 | 0.58 | 1000 |
| weighted avg | 0.59 | 0.58 | 0.58 | 1000 |

Outcome values :

291 271 180 333

|  | precision | recall | f1-score | support |
| --- | --- | --- | --- | --- |
| 0 | 0.55 | 0.65 | 0.60 | 513 |
| 1 | 0.62 | 0.52 | 0.56 | 562 |

| accuracy |  |  | 0.58 | 1075 |
| --- | --- | --- | --- | --- |
| macro avg | 0.58 | 0.58 | 0.58 | 1075 |
| weighted avg | 0.59 | 0.58 | 0.58 | 1075 |

Outcome values:

336 271 210 333

|  | precision | recall | f1-score | support |
| --- | --- | --- | --- | --- |
| 0 | 0.55 | 0.61 | 0.58 | 543 |
| 1 | 0.62 | 0.55 | 0.58 | 607 |

| accuracy |  |  | 0.58 | 1150 |
| --- | --- | --- | --- | --- |
| macro avg | 0.58 | 0.58 | 0.58 | 1150 |
| weighted avg | 0.59 | 0.58 | 0.58 | 1150 |

Outcome values:

370 271 250 334

|  | precision | recall | f1-score | support |
| --- | --- | --- | --- | --- |
| 0 | 0.55 | 0.57 | 0.56 | 584 |
| 1 | 0.60 | 0.58 | 0.59 | 641 |

| accuracy |  |  | 0.57 | 1225 |
| --- | --- | --- | --- | --- |
| macro avg | 0.57 | 0.57 | 0.57 | 1225 |
| weighted avg | 0.58 | 0.57 | 0.57 | 1225 |

Outcome values:

405 271 290 334

|  | precision | recall | f1-score | support |
| --- | --- | --- | --- | --- |
| 0 | 0.55 | 0.54 | 0.54 | 624 |
| 1 | 0.58 | 0.60 | 0.59 | 676 |

| accuracy |  |  | 0.57 | 1300 |
| --- | --- | --- | --- | --- |
| macro avg | 0.57 | 0.57 | 0.57 | 1300 |
| weighted avg | 0.57 | 0.57 | 0.57 | 1300 |

Outcome values:

443 271 327 334

|  | precision | recall | f1-score | support |
| --- | --- | --- | --- | --- |
| 0 | 0.55 | 0.51 | 0.53 | 661 |
| 1 | 0.58 | 0.62 | 0.60 | 714 |

| accuracy |  |  | 0.57 | 1375 |
| --- | --- | --- | --- | --- |
| macro avg | 0.56 | 0.56 | 0.56 | 1375 |
| weighted avg | 0.56 | 0.57 | 0.56 | 1375 |

Outcome values:

481 271 361 337

|  | precision | recall | f1-score | support |
| --- | --- | --- | --- | --- |
| 0 | 0.55 | 0.48 | 0.52 | 698 |
| 1 | 0.57 | 0.64 | 0.60 | 752 |

| accuracy |  |  | 0.56 | 1450 |
| --- | --- | --- | --- | --- |
| macro avg | 0.56 | 0.56 | 0.56 | 1450 |
| weighted avg | 0.56 | 0.57 | 0.56 | 1450 |

Outcome values:

525 271 392 337

|  | precision | recall | f1-score | support |
| --- | --- | --- | --- | --- |
| 0 | 0.55 | 0.486 | 0.50 | 729 |
| 1 | 0.57 | 0.66 | 0.61 | 796 |

| accuracy |  |  | 0.57 | 1525 |
| --- | --- | --- | --- | --- |
| macro avg | 0.56 | 0.56 | 0.56 | 1525 |
| weighted avg | 0.56 | 0.57 | 0.56 | 1525 |

Outcome values:

558 276 423 343

|  | precision | recall | f1-score | support |
| --- | --- | --- | --- | --- |
| 0 | 0.55 | 0.45 | 0.50 | 766 |
| 1 | 0.57 | 0.67 | 0.61 | 834 |

| accuracy |  |  | 0.56 | 1600 |
| --- | --- | --- | --- | --- |
| macro avg | 0.56 | 0.56 | 0.56 | 1600 |
| weighted avg | 0.56 | 0.57 | 0.56 | 1600 |

Outcome values:

582 298 433 362

|  | precision | recall | f1-score | support |
| --- | --- | --- | --- | --- |
| 0 | 0.55 | 0.46 | 0.50 | 795 |
| 1 | 0.57 | 0.66 | 0.61 | 880 |

| accuracy |  |  | 0.56 | 1675 |
| --- | --- | --- | --- | --- |
| macro avg | 0.56 | 0.56 | 0.56 | 1675 |
| weighted avg | 0.56 | 0.56 | 0.56 | 1675 |

Outcome values:

631 304 450 365

|  | precision | recall | f1-score | support |
| --- | --- | --- | --- | --- |
| 0 | 0.55 | 0.45 | 0.49 | 815 |
| 1 | 0.58 | 0.67 | 0.63 | 935 |

| accuracy |  |  | 0.57 | 1750 |
| --- | --- | --- | --- | --- |
| macro avg | 0.56 | 0.56 | 0.56 | 1750 |
| weighted avg | 0.57 | 0.57 | 0.56 | 1750 |

Outcome values:

692 304 464 365

|  | precision | recall | f1-score | support |
| --- | --- | --- | --- | --- |
| 0 | 0.55 | 0.44 | 0.49 | 829 |
| 1 | 0.60 | 0.69 | 0.64 | 996 |

| accuracy |  |  | 0.58 | 1825 |
| --- | --- | --- | --- | --- |
| macro avg | 0.57 | 0.57 | 0.57 | 1825 |
| weighted avg | 0.57 | 0.58 | 0.57 | 1825 |

Outcome values:

753 304 478 365

|  | precision | recall | f1-score | support |
| --- | --- | --- | --- | --- |
| 0 | 0.55 | 0.43 | 0.48 | 843 |
| 1 | 0.61 | 0.71 | 0.66 | 1057 |

| accuracy |  |  | 0.59 | 1900 |
| --- | --- | --- | --- | --- |
| macro avg | 0.58 | 0.57 | 0.57 | 1900 |
| weighted avg | 0.58 | 0.59 | 0.58 | 1900 |

Outcome values:

806 304 500 365

|  | precision | recall | f1-score | support |
| --- | --- | --- | --- | --- |
| 0 | 0.55 | 0.42 | 0.48 | 865 |
| 1 | 0.62 | 0.73 | 0.67 | 1110 |

| accuracy |  |  | 0.59 | 1975 |
| --- | --- | --- | --- | --- |
| macro avg | 0.58 | 0.57 | 0.57 | 1975 |
| weighted avg | 0.59 | 0.59 | 0.58 | 1975 |

Outcome values:

865 304 516 365

|  | precision | recall | f1-score | support |
| --- | --- | --- | --- | --- |
| 0 | 0.55 | 0.41 | 0.47 | 881 |
| 1 | 0.63 | 0.74 | 0.68 | 1169 |

| accuracy |  |  | 0.60 | 2050 |
| --- | --- | --- | --- | --- |
| macro avg | 0.59 | 0.58 | 0.57 | 2050 |
| weighted avg | 0.59 | 0.60 | 0.59 | 2050 |

Outcome values :

916 304 540 365

|  | precision | recall | f1-score | support |
| --- | --- | --- | --- | --- |
| 0 | 0.55 | 0.40 | 0.46 | 905 |
| 1 | 0.63 | 0.75 | 0.68 | 1220 |

| accuracy |  |  | 0.60 | 2125 |
| --- | --- | --- | --- | --- |
| macro avg | 0.59 | 0.58 | 0.57 | 2125 |
| weighted avg | 0.59 | 0.60 | 0.59 | 2125 |

Outcome values:

978 304 553 365

|  | precision | recall | f1-score | support |
| --- | --- | --- | --- | --- |
| 0 | 0.55 | 0.40 | 0.46 | 918 |
| 1 | 0.64 | 0.76 | 0.70 | 1282 |

| accuracy |  |  | 0.61 | 2200 |
| --- | --- | --- | --- | --- |
| macro avg | 0.59 | 0.58 | 0.58 | 2200 |
| weighted avg | 0.60 | 0.61 | 0.60 | 2200 |

Outcome values:

1039 304 567 365

|  | precision | recall | f1-score | support |
| --- | --- | --- | --- | --- |
| 0 | 0.55 | 0.39 | 0.46 | 932 |
| 1 | 0.65 | 0.77 | 0.70 | 1343 |

| accuracy |  |  | 0.62 | 2275 |
| --- | --- | --- | --- | --- |
| macro avg | 0.60 | 0.58 | 0.58 | 2275 |
| weighted avg | 0.61 | 0.62 | 0.60 | 2275 |

Outcome values:

1096 304 585 365

|  | precision | recall | f1-score | support |
| --- | --- | --- | --- | --- |
| 0 | 0.55 | 0.38 | 0.45 | 950 |
| 1 | 0.65 | 0.78 | 0.71 | 1400 |

| accuracy |  |  | 0.62 | 2350 |
| --- | --- | --- | --- | --- |
| macro avg | 0.60 | 0.58 | 0.58 | 2350 |
| weighted avg | 0.61 | 0.62 | 0.61 | 2350 |

Outcome values:

1151 304 605 365

|  | precision | recall | f1-score | support |
| --- | --- | --- | --- | --- |
| 0 | 0.55 | 0.38 | 0.45 | 970 |
| 1 | 0.66 | 0.79 | 0.72 | 1455 |

| accuracy |  |  | 0.63 | 2425 |
| --- | --- | --- | --- | --- |
| macro avg | 0.60 | 0.58 | 0.58 | 2425 |
| weighted avg | 0.61 | 0.63 | 0.61 | 2425 |

Outcome values:

1205 304 626 365

|  | precision | recall | f1-score | support |
| --- | --- | --- | --- | --- |
| 0 | 0.55 | 0.37 | 0.44 | 991 |
| 1 | 0.66 | 0.80 | 0.72 | 1509 |

| accuracy |  |  | 0.63 | 2500 |
| --- | --- | --- | --- | --- |
| macro avg | 0.60 | 0.58 | 0.58 | 2500 |
| weighted avg | 0.61 | 0.63 | 0.61 | 2500 |

Outcome values:

1257 304 649 365

|  | precision | recall | f1-score | support |
| --- | --- | --- | --- | --- |
| 0 | 0.55 | 0.36 | 0.43 | 1014 |
| 1 | 0.66 | 0.81 | 0.73 | 1561 |

| accuracy |  |  | 0.63 | 2575 |
| --- | --- | --- | --- | --- |
| macro avg | 0.60 | 0.58 | 0.58 | 2575 |
| weighted avg | 0.61 | 0.63 | 0.61 | 2575 |
